# Preparation and Characterization of Functionalized Surgical Meshes for Early Detection of Bacterial Infections

**DOI:** 10.1021/acsbiomaterials.2c01319

**Published:** 2023-01-24

**Authors:** Adrián Fontana-Escartín, Karima El Hauadi, Sonia Lanzalaco, Maria M. Pérez-Madrigal, Elaine Armelin, Pau Turon, Carlos Alemán

**Affiliations:** † Departament d’Enginyeria Química and Barcelona Research Center for Multiscale Science and Engineering, EEBE, Universitat Politècnica de Catalunya, C/ Eduard Maristany, 10-14, 08019Barcelona, Spain; ‡ B. Braun Surgical, S.A.U., Carretera de Terrassa 121, 08191Rubí (Barcelona), Spain; § Institute for Bioengineering of Catalonia (IBEC), The Barcelona Institute of Science and Technology, Baldiri Reixac 10-12, 08028Barcelona, Spain

**Keywords:** bacteria metabolism, conducting polymer, electrochemical sensor, NADH detection, plasma treatment, smart meshes

## Abstract

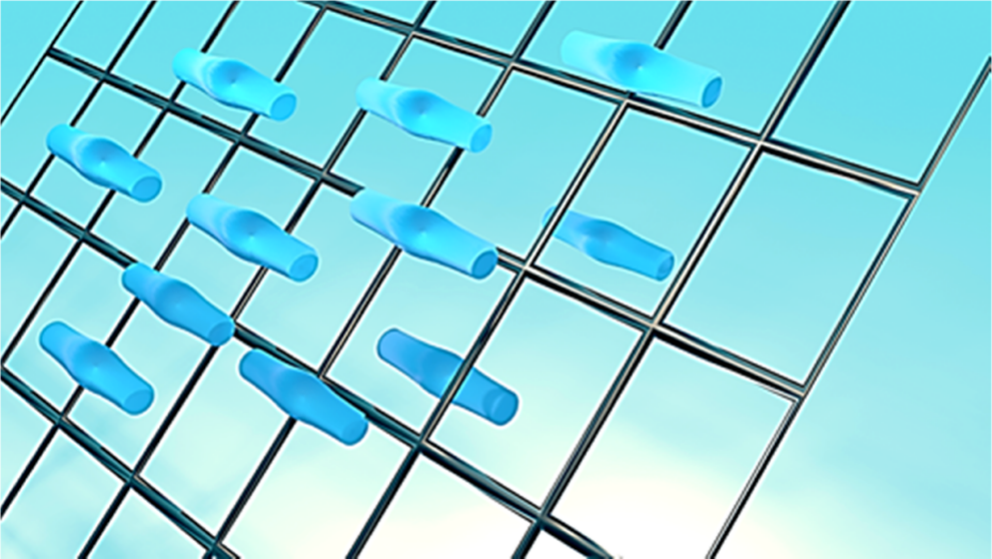

Isotactic polypropylene (i-PP) nonabsorbable surgical meshes are modified by incorporating a conducting polymer (CP) layer to detect the adhesion and growth of bacteria by sensing the oxidation of nicotinamide adenine dinucleotide (NADH), a metabolite produced by the respiration reactions of such microorganisms, to NAD+. A three-step process is used for such incorporation: (1) treat pristine meshes with low-pressure O_2_ plasma; (2) functionalize the surface with CP nanoparticles; and (3) coat with a homogeneous layer of electropolymerized CP using the nanoparticles introduced in (2) as polymerization nuclei. The modified meshes are stable and easy to handle and also show good electrochemical response. The detection by cyclic voltammetry of NADH within the interval of concentrations reported for bacterial cultures is demonstrated for the two modified meshes. Furthermore, *Staphylococcus aureus* and both biofilm-positive (B+) and biofilm-negative (B-) *Escherichia coli* cultures are used to prove real-time monitoring of NADH coming from aerobic respiration reactions. The proposed strategy, which offers a simple and innovative process for incorporating a sensor for the electrochemical detection of bacteria metabolism to currently existing surgical meshes, holds considerable promise for the future development of a new generation of smart biomedical devices to fight against post-operative bacterial infections.

## Introduction

Meshes for abdominal surgical procedures are flexible medical devices used to treat hernias, abdominal or inguinal, by adding tension-free reinforcement that results in improved tissue integration and reparation.^1,2^ Meshes have also been used as prosthetic devices for the treatment of vaginal prolapse^3,4^ and urinary incontinence.^5^ In the last decade, many fundamental research has been performed on tissue compatibility, bio-integration, and mechanical properties of surgical meshes.^6−13^ Also, their chemical surface functionalization has resulted in significant improvements, for example, prevention of unwanted tissue adhesion,^14^ cell adhesion/de-adhesion control features,^15^ and thermosensitive^16^ and shape memory^17^ properties.

Although abdominal surgical mesh implants can be prepared using a wide variety of absorbable and nonabsorbable synthetic polymers *(e.g.*, polyglycolic acid, polycaprolactone, polyethylene terephthalate, and poly(tetrafluoroethylene)), the gold-standard material of hernia meshes is isotactic polypropylene (i-PP) since its first use in the late 50s.^18,19^ Nonabsorbable i-PP warp-knitted, lightweight, and large pore-size meshes exhibit many desirable advantages, such as biocompatibility, hydrophobicity, nonimmunogenic and noncarcinogenic properties, and stability that can withstand a maximum abdominal pressure of 170 mm Hg. However, its use is not completely free from adverse factors.

The main risks of i-PP surgical meshes are that such medical devices are likely to be colonized by bacteria and further biofilm formation due to the uneven topography of knitted meshes as a consequence of a surgical site infection.^20−22^ Although bacterial infections resulting from surgical implants are rare, the importance of this drawback cannot be underestimated as they are difficult to treat, requiring long periods of antibiotic therapy and, sometimes, repeated surgical procedures.^22,23^ As bacterial colonies are mainly established in the interstices among fibers, the appropriate design of the surgical mesh is a factor to be considered to minimize the risk of bacterial infection. In addition, the root causes of infection have been deeply investigated; most likely the deficient application of aseptic protocols during the surgical procedure, long operating time, and effectiveness of antibiotic therapy combined with patient factors, such as chronic diseases, can lead to serious complications.^22^

In the first stage of mesh infection, bacteria adhere to the prosthesis-exposed surface, which is caused by the interaction between bacteria and the mesh. The progression of the bacterial colonization on the implanted foreign body leads to the formation of a bacterial biofilm. Thus, the initial bacterial adherence to the mesh, which is rapid and reversible, becomes irreversible, resulting in a biofilm after the synchronization of bacteria forming the colony that secrets an exopolysaccharide that binds and protects the colony from the external attacks.^23^ Meshes containing biofilms are resistant to both the antibiotic therapy and the host immune response, and in the most critical cases, the removal of the infected mesh is needed. By interfering with tissue integration and repair, infection has the potential to increase other significant comorbidities such as recurrence, inflammation, adhesion, and even structural loss of the abdominal wall.^24^ For these reasons, the prevention of post-surgical bacterial infection is a research hot topic.

Different strategies have been proposed to avoid post-operative mesh infection. The oldest and most conventional one is the oral and/or systemic antibiotic administration.^25^ However, considering that antibiotic overexposure predisposes to antibiotic resistance, which is a global public health problem,^26^ efforts have been focused on smarter strategies based on mesh functionalization. For example, meshes have been functionalized with plasmonic nanoparticles to eliminate biofilms by converting near-infrared light into heat,^27^ even though the most explored approach is the loading of antibiotics for sustained or controlled local release.^28−30^

In this work, we present an approach based on the early detection of bacterial colonization through the use of sensors to prevent biofilms, avoiding complex infections with long-term treatments or reoperations that could lead to the serious complications for the patient and extraordinarily high costs for the health-care system. More specifically, our strategy consists on the functionalization of surgical meshes with a sensor that was specifically developed to detect bacteria without interference of normal eukaryotic cells.^31,32^ The sensor is based on the electrochemical detection of the oxidation of nicotinamide adenine dinucleotide (NADH) into NAD+. Although NADH is involved in the respiration reactions of both bacteria and normal eukaryotic cells, a distinctive feature allows the identification of bacterial colonization without interference from signals coming from normal cells. This is that the inner membrane of mitochondria, in which the respiratory chain reactions of eukaryotic cells take place, is impermeable to NADH and NAD^+^,^33,34^ while the metabolism of bacteria occurs in the cytosol that is surrounded by the prokaryotic cell membrane, which is permeable to NADH and NAD+.^35^ Accordingly, in bacteria, the NADH and NAD+ migrate to the extracellular space, whereas in conventional cells, they remain in the cytosolic pool. The electrochemical response of the modified meshes toward NADH coming from bacterial respiration reactions demonstrates that the proposed strategy can be used to develop smart medical devices that, in addition to supporting weakened or damaged tissue, are also capable of detecting intra- and post-operative bacterial infections.

## Methods

### Materials

Lithium perchlorate (LiClO_4_), hydroxymethyl-3,4-ethylenedioxythiophene (HEDOT; 95%), 3,4-ethylenedioxythiophene (EDOT; 97%), acetonitrile (99.8%), and phosphate-buffered saline (PBS) solution were purchased from Sigma-Aldrich. LiClO_4_ was stored in an oven at 80 °C before its use in the anodic polymerization. Ammonium persulfate (APS; 98%), hydrochloric acid (37%), and sodium hydroxide were used as received from Panreac Quimica S.A.U. (Spain).

Monofilament, sterilized, and i-PP meshes, which were provided by B. Braun Surgical S.A.U. (Rubí, Spain), were used for this work. These consisted of Optilene mesh LP (OMLP) and Optilene mesh elastic (OME). OMLP is a lightweight (around 36 g/m^2^) mesh with 0.39 mm of thickness and 1 mm of pore diameter, while OME is a lightweight (around 48 g/m^2^) mesh with multidirectional elasticity, 0.55 mm of thickness, and 3.6 × 2.8 mm pore size.

### Integration of the Bacteria Sensor in Surgical Meshes

**Figure 1:**
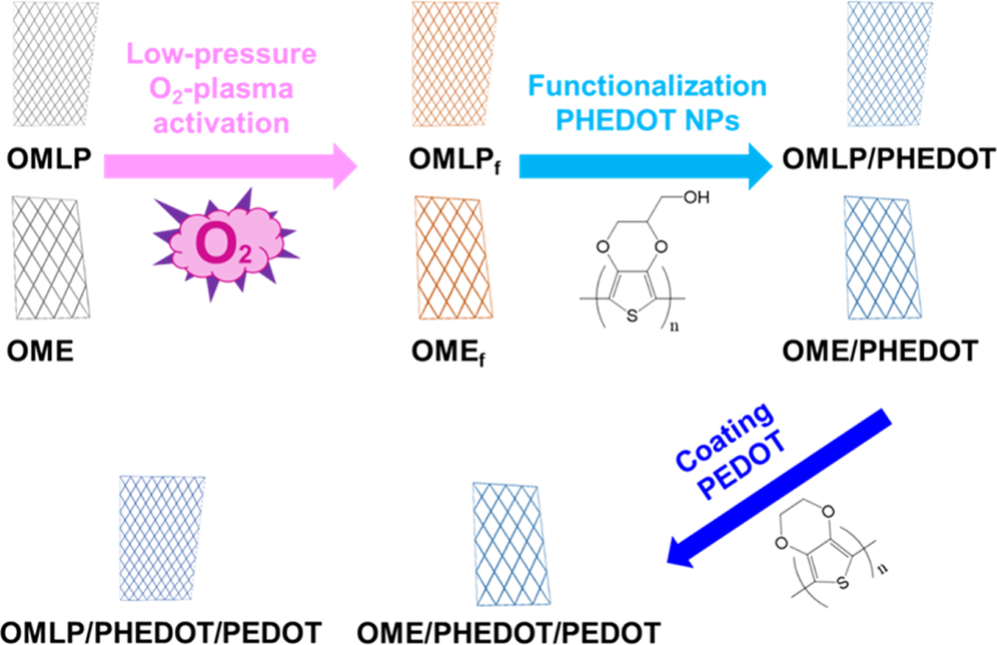
Process used to prepare OMLPf/PHEDOT/PEDOT and OMEf/PHEDOT/PEDOT meshes using OMLP and OME as starting materials.

The three-step process used to modify OMLP and OME meshes (Figure 1) consists of (1) plasma activation; (2) functionalization with poly(hydroxymethyl-3,4-ethylenedioxythiophene) nanoparticles (PHEDOT NPs), which were synthesized by chemical oxidative polymerization; and (3) coating of the functionalized meshes with a poly(3,4-ethylenedioxythiophene) (PEDOT) layer prepared by anodic polymerization. It is worth noting that PHEDOT and PEDOT are biocompatible conducting polymers,^36−40^ which are not expected to alter the biocompatibility of the i-PP used to fabricate OMLP and OME meshes.^41^

#### Plasma Activation of i-PP Meshes

The surface of OMLP and OME meshes was activated with low-pressure radio-frequency (RF) plasma (80 MHz), using a LFG generator 1000 W (Diener Electronic GmbH Co., Germany) with a reactor chamber of 25 dm^3^. For this purpose, i-PP meshes were cut in pieces of 10 × 10 cm^2^, which were placed on a glass support and, subsequently, introduced inside the chamber. After purging to eliminate the air, the chamber was filled with oxygen until a final pressure of 0.33 mbar. Once the pressure was reached, the plasma was applied for 180 s. The power discharge was 250 W. To avoid air contamination, after the plasma treatment, the samples were stored in a vacuum bag. Hereafter, the plasma-activated OMLP and OME meshes will be denoted OMLP_f_ and OME_f_, respectively.

#### Functionalization with Poly(hydroxymethyl-3,4-ethylenedioxythiophene) Nanoparticles

PHEDOT NPs were adhered to the plasma-activated meshes using a chemical oxidative polymerization process. The OMLP_f_ and OME_f_ meshes were cut in 0.5 × 1.5 cm^2^ samples and immersed by pairs in 1 mL of a 0.2 M HCl solution with 50 mM HEDOT monomer for 30 min at room temperature under stirring (250 rpm). After this, 0.2 mL of a 0.2 M HCl solution with 60 mM APS was slowly dropped into the solution containing the plasma-functionalized samples. The oxidative polymerization reaction was maintained for 24 h at 37 °C under agitation (80 rpm). After such time, the samples were removed from the reaction medium, washed three times with milli-Q water, once with acetone, and dried at room temperature.

#### Chemical Polymerization of Poly(3,4-ethylenedioxythiophene)

Functionalized meshes were immersed in a solution containing 2 mL of ethanol, 38.8 μL of EDOT monomer, and 0.0226 g of FeCl_3_. Samples were kept in an open Eppendorf to allow ethanol evaporation and, therefore, the deposition of PEDOT obtained by oxidative polymerization.

#### Electrochemical Polymerization of Poly(3,4-ethylenedioxythiophene)

OMLP_f_/PHEDOT and OME_f_/PHEDOT meshes were coated with a layer of PEDOT, which was generated by *in situ* electrochemical polymerization of EDOT onto the surface of the chemically polymerized samples. For this purpose, meshes were previously washed with 0.2 M NaOH to balance the charge. EDOT was polymerized by chronoamperometry (CA) under a constant potential of +1.40 V, adjusting the polymerization charge. The effects of the mesh geometry in the adjustment of the polymerization charge and the choice EDOT monomer concentration were carefully evaluated, as is discussed in the next section. Polymerizations were carried out with a VersaStat II potentiostat-galvanostat controlled by a Power Suite Princeton Applied Research program. The setup consisted of an electrochemical cell filled with 10 mL of an acetonitrile solution containing a given amount (10, 25 or 50 mM) of EDOT monomer and 0.1 M LiClO_4_, as a supporting electrolyte. The meshes (0.5 × 1.5 cm^2^) were employed as a working electrode, whereas a platinum rod and a Ag|AgCl electrode were used as counter and reference electrodes. PEDOT-coated meshes were washed three times with milli-Q water and dried at room temperature.

### Chemical and Structural Characterization

Scanning electron microscopy (SEM) was performed using a Focused Ion Beam Zeiss Neon40 scanning electron microscope operating at 5 kV and equipped with an energy-dispersive X-ray analysis (EDX) spectroscopy system. The size of the nanoparticles and the diameter of the monofilaments were measured with the SmartTiff software from Carl Zeiss SMT Ltd.

Atomic force microscopy (AFM) images were taken with a Molecular Imaging PicoSPM and a NanoScope IV controller, under ambient conditions. The AFM tapping mode was operated at constant deflection. The row scanning frequency was set to 1 Hz. AFM measurements were performed on various parts of the meshes, which provided reproducible images. The scan window sizes used were 10 × 10 μm^2^. The statistical application of the NanoScope Analysis software was used to determine the root mean square roughness (*R*_q_), which is the average height deviation taken from the mean data plane.

Fourier-transform infrared spectroscopy (FTIR) spectra were acquired using a Jasco 4100 spectrophotometer equipped with an attenuated total reflection accessory (Top-plate) with a diamond crystal (Specac model MKII Golden Gate Heated Single Reflection Diamond ATR) reflectance standard. Samples were evaluated using the spectra manager software, and for each sample, 64 scans were performed between 4000 and 600 cm^–1^ with a resolution of 4 cm^–1^.

Samples were characterized by micro-Raman spectroscopy using a commercial Renishaw inVia Qontor confocal Raman microscope. The Raman setup consisted of a laser (at 785 nm with a nominal 300 mW output power) directed through a microscope (specially adapted Leica DM2700 M microscope) to the sample after which the scattered light is collected and directed to a spectrometer with a 1200 lines·mm^–1^ grating. The exposure time was 10 s, the laser power was adjusted to 1% of its nominal output power, and each spectrum was collected with three accumulations.

### Electrochemical Characterization and Detection of NADH

Electrochemical assays were conducted using cyclic voltammetry (CV). Characterization of the coated meshes was performed using a three-electrode cell and an Autolab PGSTAT302N and NOVA software. The coated meshes and a platinum wire were employed as working and counter electrodes, respectively, while the reference electrode was an Ag|AgCl electrode containing a potassium chloride (KCl) saturated aqueous solution (*E*^0^ = 0.222 V at 25 °C). The initial and final potentials were −0.20 V, and the reversal potential was +0.80 V.

The electrochemical detection of ferricyanide, Fe(CN)_6_^3–^, was performed using the coated meshes as a working electrode and the combined Pt//Ag|AgCl electrode. Ten milliliters of a 0.1 M with different concentrations of Fe(CN)_6_^3–^ (from 0 to 1.0 mM) was introduced in a cell, and the detection was performed using CV. Similarly, the detection of NADH was studied by CV, even though in this case we used separated electrodes (Pt wire as counter electrode and Ag|AgCl as reference electrode). Measurements were performed by adding different concentrations of NADH (from 0 to 6 mM) to 5 mL of the electrolytic medium, which was a 0.1 M PBS solution. In all cases, the initial and final potentials were −0.20 V, and the reversal potential was +0.80 V.

The porosity was indirectly quantified through the parameter Δ^42^
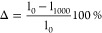
1where  and  refer to the thickness before applying any oxidation–reduction cycle (*n*_redox_= 0) and after 1000 consecutive oxidation–reduction cycles (*n*_redox_= 1000) in an acetonitrile solution containing 0.1 M LiClO_4_, which is a much less aggressive medium than PBS. This procedure is based on the fact that the degree of compactness induced by consecutive redox cycles depends on the porosity of the fresh sample, both the degree of compactness and porosity of the films being related to their thickness.

### Electrochemical Detection of NADH in Bacteria Culture Medium Solutions

A 2 x 10^8^ CFU/mL of *Staphylococcus aureus* (*S. aureus*) and biofilm-positive (B+) and biofilm-negative (B-) *Escherichia coli* (*E. coli*) strains were seeded in 10 mL of Dulbecco’s Modified Eagle Medium (DMEM) supplemented with 2% FBS (fetal bovine serum) and 0.2% NaHCO_3_ (pH adjusted at 7.4). After 24 h at 37 °C and 80 rpm, the same amount of CFU/mL was added to 500 mL of the same supplemented medium. Cultures were maintained at 37 °C and 80 rpm for 24 h more, thus promoting bacteria growth (absorbance values between 1.6–1.8 at 600 nm). Controls were prepared in 500 mL of DMEM supplemented without bacteria but under the same culture conditions. Then, all solutions (*i.e.*, the control, *S. aureus*, B+ E. coli, or B– *E. coli* cultures, here labeled as 1×) were centrifuged at 10^4^ rpm and 4 °C for 10 min. The supernatant was frozen in liquid nitrogen and lyophilized until dry. The resulting powders were dissolved in 20 mL of Milli-Q water, becoming equivalent to 19×. The 19× solution from *S. aureus*, B+ *E. coli*, or B– *E.coli* were consecutively diluted, retaining in all cases 5 mL to perform electrochemical detection. The electrochemical detection of NADH concentration values was followed by CV using a three-electrode cell and an Autolab PGSTAT302N and NOVA software. The functionalized meshes and platinum wire were employed as working and counter electrodes, respectively, while the reference electrode was an Ag|AgCl electrode containing *a* KCl-saturated aqueous solution (*E*^0^ = 0.222 V at 25 °C). The initial and final potentials were −0.20 V, and the reversal potential was +0.80 V. To obtain a calibration curve, which allowed us to convert current into NADH concentration values, measurements were performed by adding different known NADH concentration values (*i.e.*, from 0 to 8 mM) to the control bacteria culture medium, which was DMEM supplemented without bacteria at 1× before freeze-drying.

### Statistical Analysis

All experiments were performed in triplicate. Results are expressed as the mean ± standard deviation.

## Results and Discussion

### Characterization of the Functionalized Surgical Meshes

Low-pressure oxygen plasma treatment was applied to OMLP and OME meshes (10 × 10 cm^2^ samples) using the conditions described in the Methods section, the plasma-activated meshes being denoted OMLP_f_ and OME_f_, respectively. The choice of the conditions for the plasma activation was based on a previous study in which the plasma treatment procedure was optimized for the subsequent functionalization of OMLP meshes with a thermoresponsive hydrogel.^43^ The effect of plasma on the surface morphology and topography of the i-PP fibers was moderate, as it was characterized by scanning electron microscopy (SEM) and atomic force microscopy (AFM) (Figures S1 and S2). Thus, the surface morphology did not show any appreciable change, while the surface roughness increased from *R*_q_ = 32.8 ± 3.4 nm to *R*_q_ = 49.4 ± 5.6 nm after activation.

**Figure 2:**
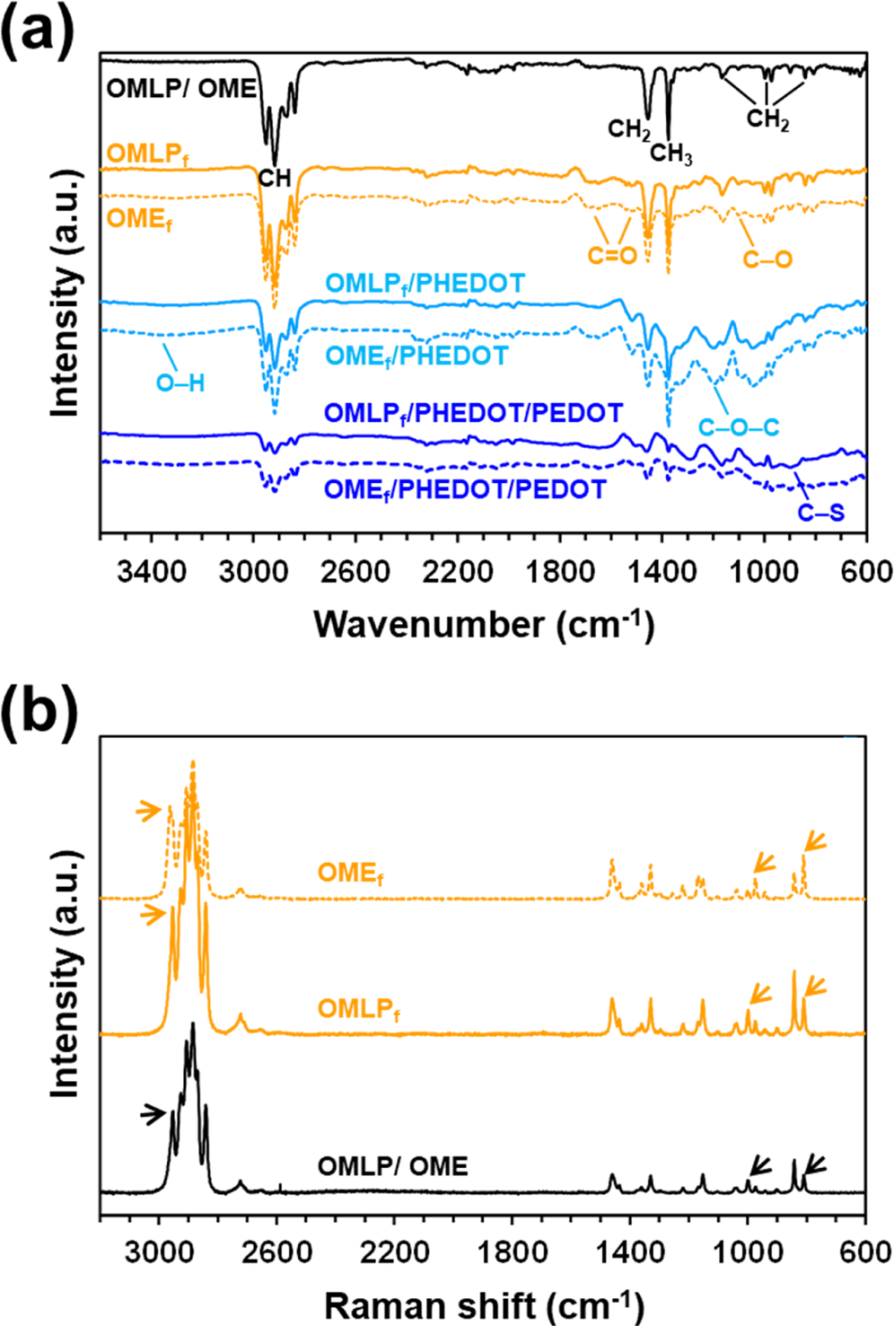
(a) FTIR spectra of pristine, activated, functionalized, and coated meshes. (b) Raman spectra of the pristine and activated meshes (comparison with functionalized and coated meshes is shown in Figure S3). Arrows indicate the bands that increased due to plasma activation.

FTIR spectra of pristine and plasma-activated meshes are compared in Figure 2a. The characteristic peaks of i-PP,^31^ which are detected in spectra of the OMLP and OME meshes, are listed in Table S1. The creation of oxygen-containing functional groups through plasma treatment was confirmed by the appearance of the C=O and the C–O stretching vibrations in the spectra of OMLP_f_ and OME_f_ (Table 1). As the less altered site after plasma activation was the CH stretching, the intensity of the peak at 2915 cm^–1^ will be used as a reference to study the modification processes involving the mesh functionalization with PHEDOT NPs and its further coating with PEDOT.

**Table 1:** Main FTIR Fingerprints of the Meshes Studied in this Work

system	FTIR fingerprints
OMLP and OME	C–H stretching at 2915 cm^–1^
	CH_3_ vibration at 1376 cm^–1^
	CH_2_ unsaturation and deformation vibrations at 841, 999, 1167, and 1455 cm^–1^
OMLP_f_ and OME_f_	C=O stretching vibrations at 1534 and 1686 cm
	C–O stretching at 1088 cm^–1^
OMLP_f_/PHEDOT and OME_f_/PHEDOT	O–H stretching vibration at 3400 cm^–1^
	C–O–C bending the peak at 1189 cm^–1^
OMLP_f_/PHEDOT/PEDOT and OME_f_/PHEDOT/PEDOT	C–S vibrations of the thiophene ring at 869 cm^–1^

The changes induced by the oxygen plasma treatment in the surface of the i-PP meshes can be also analyzed using Raman spectroscopy.^15,16^ Analyses of the recorded spectra, which are displayed in Figures S3 and 2b, show that the intensity of i-PP crystalline peaks at 809 and 973 cm^–1^ and, especially, the band associated with methyl C–H stretching vibrations at 2962 cm^–1^ (marked with arrows) increased notably, as well as the band assigned to methyl C–H stretching vibrations at 2962 cm^–1^. Overall, the plasma activation step affects mainly the lateral methyl group. After plasma treatment, the 10 × 10 cm^2^ activated samples were stored in a vacuum to preserve the created functional groups from the interaction with air.

The oxygen-functional groups created on the surface of i-PP OMLP and OME meshes were employed as suitable sites for the grafting of PHEDOT NPs using a chemical oxidative polymerization process. The successful functionalization of OMLP_f_ and OME_f_ was proved by FTIR spectroscopy (Figure 2a), the meshes functionalized with PHEDOT NPs being denoted OMLP_f_/PHEDOT and OME_f_/PHEDOT, respectively. Table 1 lists the main FTIR fingerprints of the meshes with PHEDOT NPs, which are the O–H stretching vibration and the C–O–C bending. Also, a soft decrease in the intensity of the C–H stretching vibration of CH_3_ was observed, which confirms once again that the most modified component by the plasma activation and the chemical functionalization was the side methyl group. Additionally, in the Raman spectra, the C=C symmetrical stretching at 1420 cm^–1^ and the C=C asymmetrical stretching at 1507 cm^–1^ of PHEDOT were detected (Figure S3). Furthermore, the intensity of the peak corresponding to the C–H stretching of the CH_3_ group (2962 cm^–1^) decreases noticeably, which further evidences the grafting of PHEDOT NPs.

**Figure 3:**
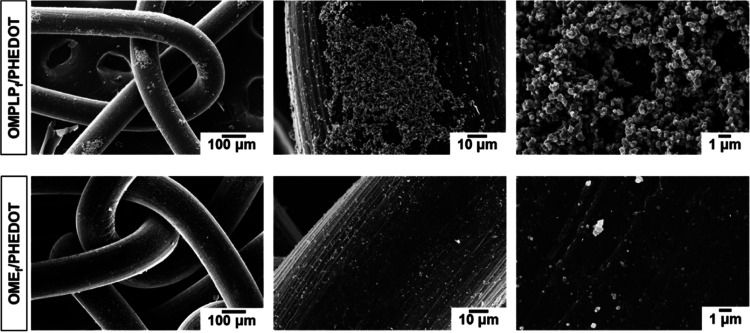
SEM micrographs of OMLP_f_/PHEDOT and OME_f_/PHEDOT.

SEM micrographs show the morphological differences between OMLP_f_/PHEDOT and OME_f_/PHEDOT meshes (Figure 3). PHEDOT NPs formed heterogeneous aggregates on the surface of OMLP_f_/PHEDOT, while the NPs were homogeneously dispersed on the surface of the OME_f_/PHEDOT mesh. Furthermore, the average size of PHEDOT NPs was 220 ± 40 nm (from 119 to 288 nm) and 140 ± 28 nm (from 96 to 200 nm) for OMLP_f_/PHEDOT and OME_f_/PHEDOT, respectively. The differences in the grafting of the CP NPs was attributed to several factors: (i) the different monofilament thickness, which was 120 ± 1 μm and 153 ± 1 μm for OMLP_f_/PHEDOT and OME_f_/PHEDOT, respectively, (ii) the porosity, and (iii) the pattern of crossing in the meshes, which undoubtedly affected their activation by plasma.

In the last step of the sensor preparation process, the functionalized meshes were coated with PEDOT (Figure 1). Due to the complex geometry of the meshes, two different strategies were considered: oxidative polymerization and electrochemical polymerization. In the first strategy, the meshes were immersed in an ethanol solution containing EDOT monomer and FeCl_3_ for oxidative polymerization. Unfortunately, the coating process by strategy was unsuccessful since the PEDOT layer was not uniformly deposited (even after 3 days) and was not well adhered to the mesh (*i.e.*, parts of the coating came off when handling the meshes).

The second strategy consisted of the electropolymerization of EDOT monomer under a constant potential. Although the electrochemical strategy was successful in the preliminary assays, it was largely improved in terms of both coating uniformity and stability when the meshes were immobilized with a stainless steel clamp, which kept the mesh as close as possible to the platinum counter electrode and oriented toward it. As the metallic clamp was partially immersed in the reaction medium, the oxidation potential required for EDOT electropolymerization was the lowest on the stainless steel surface. Thus, the metallic clamp offered a nucleation site for an EDOT monomer to start the electropolymerization, and once the first PEDOT chains were grown, the process propagated toward the mesh. Hereafter, the coated meshes are denoted OMLP_f_/PHEDOT/PEDOT and OME_f_/PHEDOT/PEDOT.

**Figure 4:**
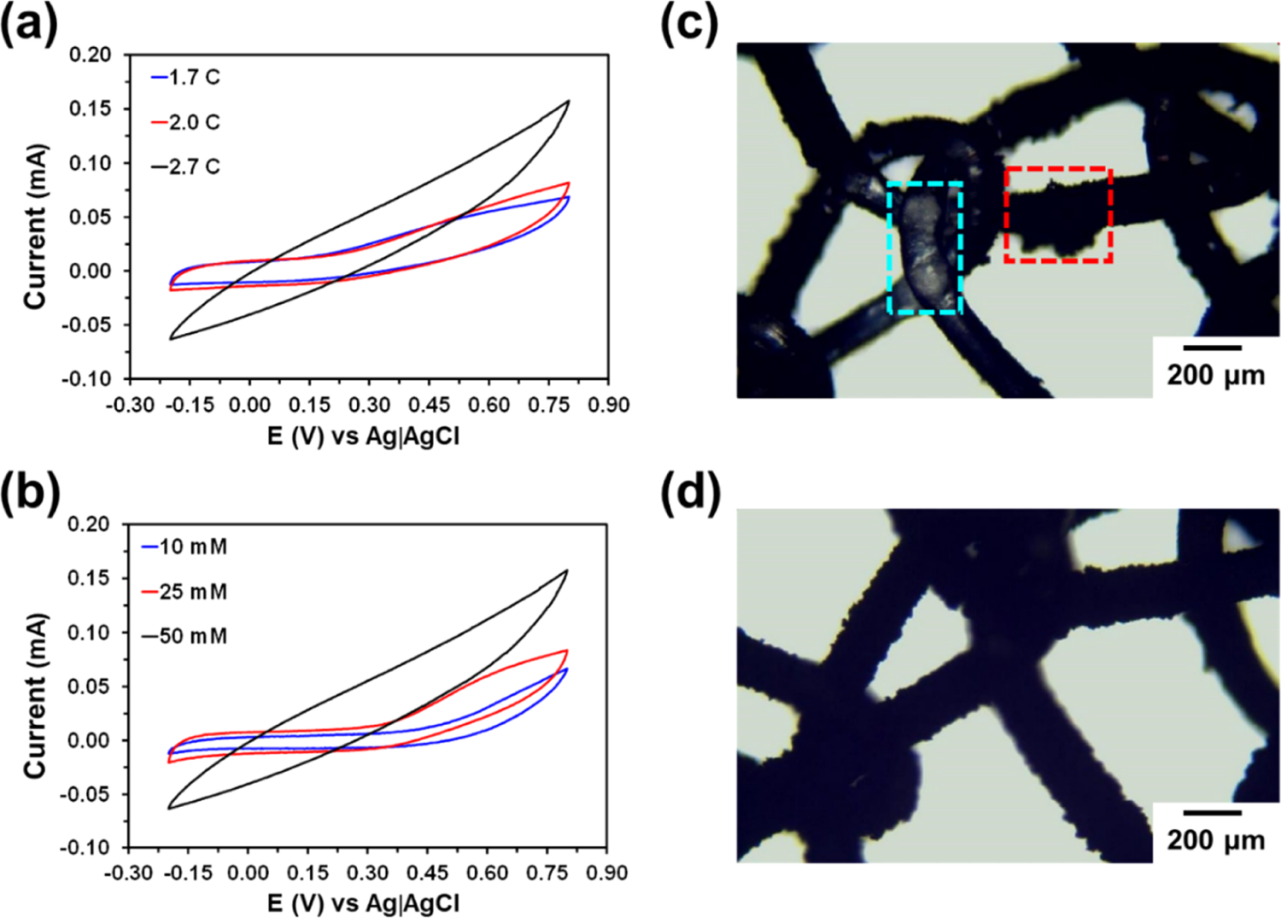
Cyclic voltammograms of OMLP_f_/PHEDOT/PEDOT meshes prepared using (a) 50 mM EDOT solution and different polymerization charges (1.7, 2.0, and 2.7 C) and (b) polymerization charge of 2.7 C and different EDOT concentrations (10, 25, and 50 mM). The voltammograms displayed correspond to the 10th consecutive redox cycle and were recorded at a scan rate of 50 mV/s. Optical micrograph of OMLP_f_/PHEDOT/PEDOT meshes prepared using (c) 50 mM EDOT concentration and a polymerization charge of 2.7 C, where the dashed red and blue boxes illustrate regions with an accumulation of PEDOT and a poor EDOT polymerization, respectively, and (d) 25 mM EDOT concentration and a polymerization charge of 2.7 C, which shows a uniform PEDOT coating.

Two operational parameters, the polymerization charge and the EDOT concentration, were optimized to achieve the maximum electrochemical response. First, the polymerization charge of EDOT was adjusted to 1.7, 2.0, or 2.7 C. The coating process by electropolymerizing an EDOT monomer at a constant potential took around 10–15 min, depending on the choice of the polymerization charge. Cyclic voltammograms in 0.1 M PBS are compared in Figure 4a. As it can be seen, the best electrochemical performance was achieved at the highest polymerization charge (2.7 C), reaching the maximum current intensity (*I*_max_) at the reversal potential, *I*_max_ = 0.16 mA. The *I*_max_ values obtained for the meshes coated using a polymerization charge of 2.0 and 1.7 C were 0.08 and 0.07 mA, respectively.

After evaluation of the influence of the polymerization charge on the electrochemical response of OMLP_f_/PHEDOT/PEDOT, the effect of the monomer concentration was investigated. For this purpose, the cell was filled with 10 mL of a 10, 25, or 50 mM monomer solution in acetonitrile with 0.1 M LiClO_4_, as a supporting electrolyte, and the polymerization charge was adjusted to 2.7 C. Results, which are shown in Figure 4b, evidenced that the response of the meshes coated using a 50 mM EDOT solution was more advantageous (*I*_max_ = 0.16 mA) than that of meshes coated using 10 and 25 mM solutions (*I*_max_ = 0.07 and 0.08 mA, respectively). However, inspection of the optical micrographs recorded for the OMLP_f_/PHEDOT/PEDOT meshes prepared using a 50 mM EDOT solution and a polymerization charge of 2.7 C revealed a nonuniform distribution of the PEDOT coating, with areas of remarkable PEDOT accumulation and regions where the PHEDOT coating was still visible (Figure 4c). This nonhomogeneous distribution of the CP was found to be unfavorable for the stability and handling of the coated mesh in the detection stage. Conversely, not only did the utilization of a 25 mM EDOT solution and a polymerization charge of 2.7 C result in a uniform PEDOT coating (Figure 4d), but also in a stable and easy-to-handle mesh. Accordingly, OMLP_f_/PHEDOT/PEDOT meshes for bacterial detection were prepared using a 25 mM EDOT solution and a polymerization charge of 2.7 C.

Unfortunately, the operational parameters selected for OMLP_f_/PHEDOT/PEDOT could not be applied to OME_f_/PHEDOT since they induced the formation of a heterogeneous PEDOT coating on the functionalized mesh surface. Accordingly, a systematic analysis similar to that displayed in Figure 4 for OMLP_f_/PHEDOT/PEDOT was conducted for OME_f_/PHEDOT/PEDOT (Figure S4). Indeed, OME_f_/PHEDOT/PEDOT meshes prepared using a 25 mM EDOT concentration and a polymerization charge of 1.7 C presented the best balance among electrochemical response, homogeneity of the coating, and handling capacity (Figure S4c).

**Figure 5:**
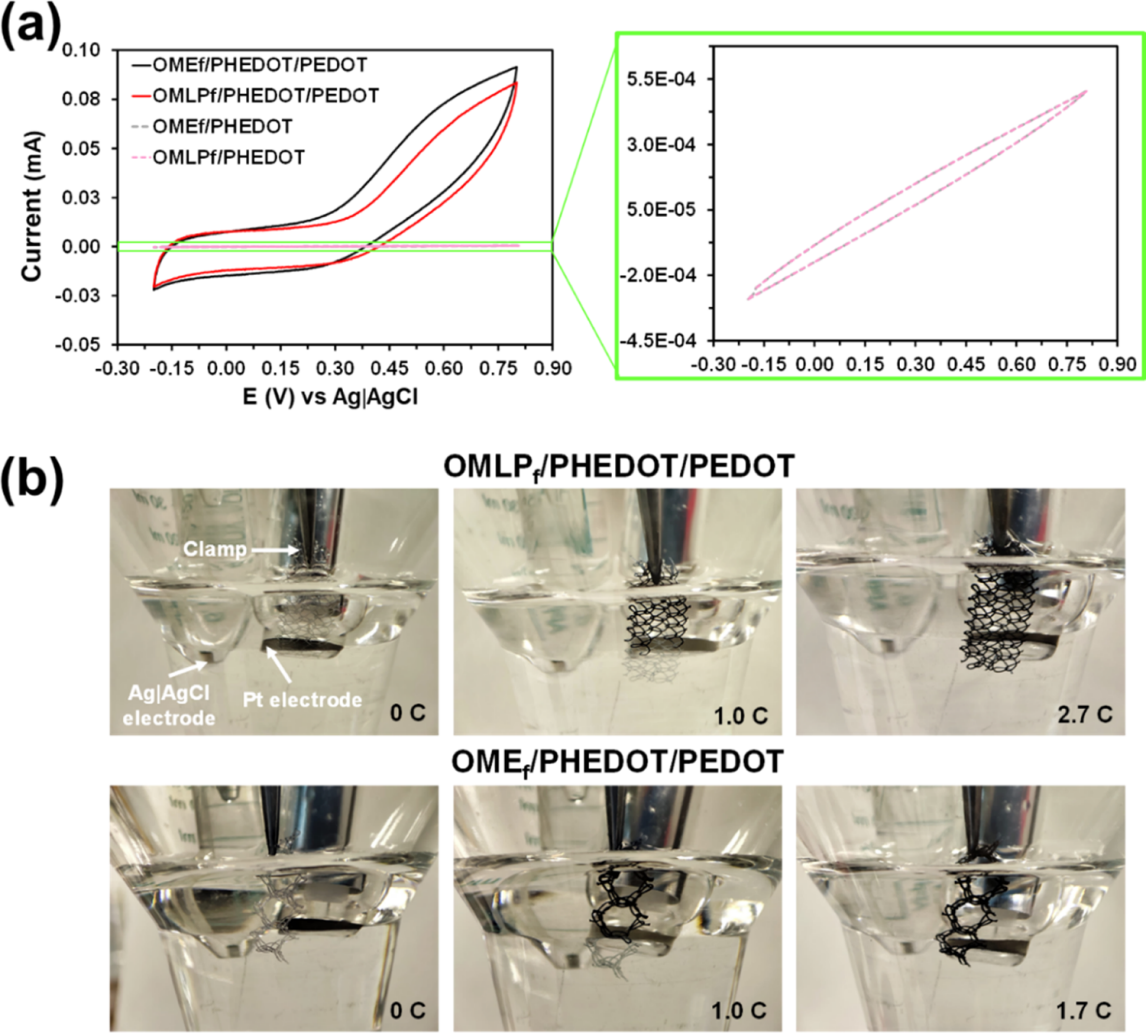
(a) Cyclic voltammograms comparing the electrochemical response of OMLP_f_/PHEDOT/PEDOT, OME_f_/PHEDOT/PEDOT, OMLP_f_/PHEDOT, and OME_f_/PHEDOT. Coated meshes were prepared using the optimized operational parameters (see text). Scan rate: 50 mV/s. (b) Photographs showing the progression of the electrochemical polymerization of a 25 mM EDOT solution on OMLP_f_/PHEDOT and OME_f_/PHEDOT. The meshes progressively change color from gray to dark blue when the polymerization charge increased from 0 C to 2.7 C (OMLP_f_/PHEDOT/PEDOT) or 1.7 C (OME_f_/PHEDOTPEDOT).

Noteworthy, the significant effect of the coating PEDOT layer in the electrochemical response of the meshes was established by comparing the cyclic voltammograms of the meshes functionalized with PHEDOT NPs before and after the coating process (Figure 5a). While OMLP_f_/PHEDOT and OME_f_/PHEDOT exhibit very low current values (*i.e., I*_max_ = 5 × 10^–4^ mA for both functionalized meshes), the current values at the reversal potential of OMLP_f_/PHEDOT/PEDOT and OME_f_/PHEDOT/PEDOT are two orders of magnitude greater (*i.e., I*_max_ = 0.09 and 0.08 mA, respectively). Hence, PEDOT creates conduction paths, which are connected by the dispersed PHEDOT NPs that act as secondary nucleation sites. Figure 5b shows the progressive formation of the PEDOT coating using the operational parameters optimized for the electropolymerization process in each mesh. As it can be seen, the PEDOT layer was first generated on the surface of the metallic clamp (first step) and then it propagated to the mesh.

On the other hand, the values of Δ (eq 1) indicate that the porosity is slightly lower for the PEDOT coating in OMLP_f_/PHEDOT/PEDOT (Δ = 34 ± 4%) than for OME_f_/PHEDOT/PEDOT (Δ = 41 ± 2%), which is fully consistent with the areas of the voltammograms displayed in Figure 5a. The porosity is intimately related to the ability to store charge, which essentially depends on the mobility of counteranions during the oxidation and reduction cycles (*i.e.*, entrance into the CP matrix and escape from the CP matrix, respectively). Thus, the higher mobility of the counterions corresponds to the materials with higher electrochemical activity (*i.e.*, higher area of the voltammogram). Besides, it should be noted that the Δ values determined in this work are similar to those reported for PEDOT directly electropolymerized on metallic electrodes (*i.e.*, steel).^42^

**Figure 6:**
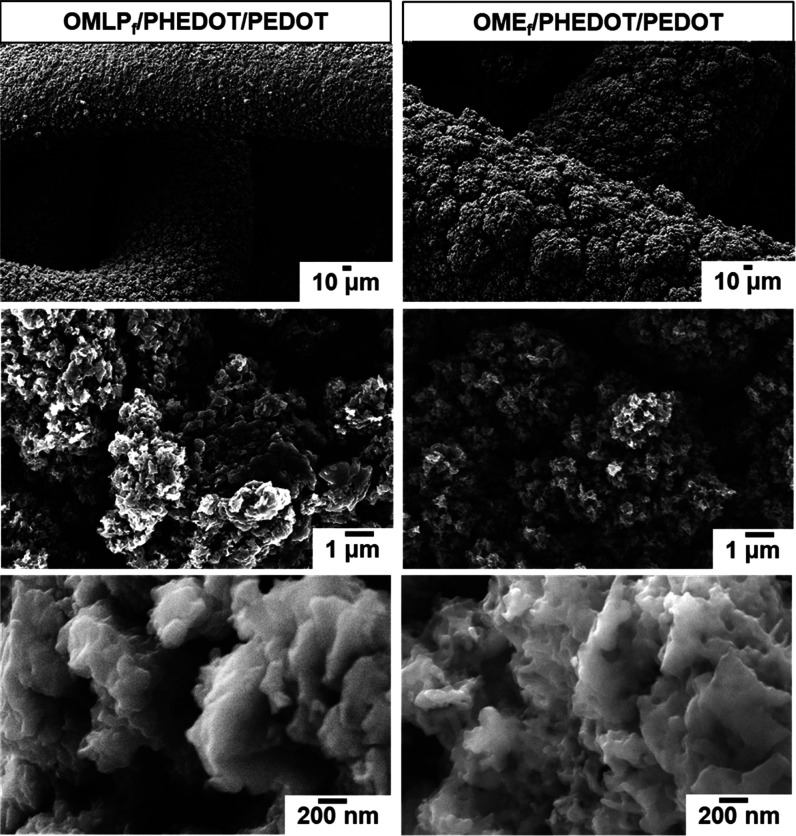
SEM micrographs of OMLP_f_/PHEDOT/PEDOT and OME_f_/PHEDOT/PEDOT.

The FTIR spectra of OMLP_f_/PHEDOT/PEDOT and OME_f_/PHEDOT/PEDOT prepared using the optimized conditions are included in Figure 2a, and the position of the peak associated with the C–S vibrations listed in Table 1.^31^ However, the most remarkable feature is that the intensity of the C–H band in the coated meshes decreases considerably with respect to the meshes functionalized with PHEDOT NPs, reflecting that the amount of CP is much higher in the former than in the latter. Besides, in the Raman spectra (Figure S3), not only did the C–H peak at 2962 cm^–1^ completely disappeared but also the intensity of the symmetric and asymmetric C=C stretching at 1420 and 1507 cm^–1^, respectively, increased. Besides, both meshes were coated by a homogeneous PEDOT layer made of uniformly distributed and leveled CP aggregates, as it was shown in the SEM micrographs of OMLP_f_/PHEDOT/PEDOT and OME_f_/PHEDOT/PEDOT (Figure 6). High-resolution SEM images indicate that the texture of the PEDOT layer at the nanometric scale is rough, which increases the active surface for electrochemical detection compared to a smooth surface.

**Figure 7:**
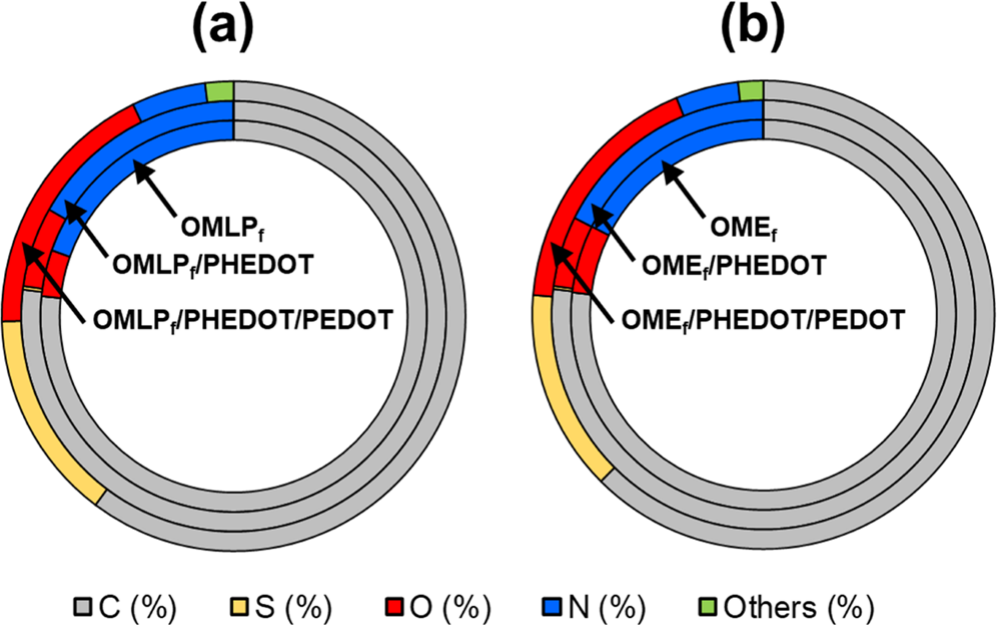
Elemental composition (in %) of activated (OMLP_f_ and OME_f_), functionalized (OMLP_f_/PHEDOT and OME_f_/PHEDOT), and coated (OMLP_f_/PHEDOT/PEDOT and OME_f_/PHEDOT/PEDOT) meshes derived from (a) OMLP and (b) OME.

In addition to FTIR and Rama spectroscopy, the success of the activation, functionalization, and coating steps was analyzed by semiquantitative SEM-EDX spectroscopy. Figure 7 displays the elemental composition of activated, functionalized, and coated OMLP and OME meshes, while the represented values are listed in Table S1. The content of oxygen, which was not detected in untreated OMLP and OME, was found to be around 4–5% for OMLP_f_ and OME_f_, increasing progressively up to ∼6 and ∼18% after the incorporation of the PHEDOT NPs and the PEDOT layer, respectively. Besides, the amount of sulfur was very low for OMLP_f_/PHEDOT and OME_f_/PHEDOT (0.3 and 0.2%, respectively), increasing up to 14.5 and 13.9% for OMLP_f_/PHEDOT/PEDOT and OME_f_/PHEDOT/PEDOT, respectively. The increment in the sulfur content after the coating step reflects the importance of the PEDOT layer and is fully consistent with the different electrochemical responses found for functionalized and coated meshes (Figure 5a).

The coating yield (CY, in mg of PEDOT / cm^2^) for OMLP_f_/PHEDOT/PEDOT and OME_f_/PHEDOT/PEDOT prepared using the optimized conditions was determined using the following expression
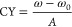
2where ω and ω_0_ are the weight after and before the coating process, respectively, and *A* is the area of the nonfunctionalized and noncoated fibers. The value of *A* was estimated using the ImageJ software and SEM micrographs. The resulting values (Table 2) indicate that the CY is ∼16% higher for OMLP_f_/PHEDOT/PEDOT than for OME_f_/PHEDOT/PEDOT, which is consistent with the elemental composition reported in Table S1 (*i.e.*, the content of sulfur was higher for the former than for the latter). This result has been attributed to the fact that the polymerization charge was greater for OMLP_f_/PHEDOT/PEDOT than for OME_f_/PHEDOT/PEDOT. Finally, Figure S5 displays photographs of the meshes after their treatment with plasma, PHEDOT functionalization, and PEDOT coating, which show that their color changed from gray to dark blue.

**Table 2:** Coating Yield (CY, Expressed as mg of PEDOT per cm^2^; Equation 2) for the Coated Meshes (*n* = 3)

mesh	polymerization charge (C)	*A* (cm^2^)	CY (mg/cm^2^)
OMLP_f_/PHEDOT/PEDOT	2.7	0.99	2.11 ± 0.10
OME_f_/PHEDOT/PEDOT	1.7	0.76	1.82 ± 0.25

### Performance of Functionalized Surgical Meshes for Electrochemical Detection

The capacity of OMLP_f_/PHEDOT/PEDOT and OME_f_/PHEDOT/PEDOT meshes to act as working electrodes is proved in Figure S6, which shows their voltammetric responses in a 0.1 M PBS solution with different concentrations of ferricyanide, Fe(CN)_6_^3–^. The latter compound is a common redox probe to test the performance of novel materials as working electrodes^44−46^
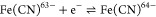
3Results evidence a significant increase in the current density and the apparition of the oxidation peaks. Both OMLP_f_/PHEDOT/PEDOT and OME_f_/PHEDOT/PEDOT meshes are able to act as an electrochemical sensor, holding oxidation reactions. However, the complex geometry of the meshes, in particular of OME_f_/PHEDOT/PEDOT, suggests that the electrochemical detection of NADH requires optimization of the conditions to avoid the shift of the oxidation peak with the analyte concentration, as occurred in Figure S6 with Fe(CN)_6_^3–^.

Results for the electrochemical detection of NADH, which was carried out by CV, are displayed in Figure S7a,b. As it can be seen, the current density at the reversal potential (*j*_max_) increased with the concentration of NADH, which according to previous observations was ascribed to the electrocatalyzed oxidation of NADH to NAD+.^31^ However, this variation was more evident for OMLP_f_/PHEDOT/PEDOT than for OME_f_/PHEDOT/PEDOT, which has been attributed to the complex geometry of the latter mesh. Furthermore, voltammograms are not symmetric, which is especially evident for OMLP_f_/PHEDOT/PEDOT.

The calibration plots, which are shown in Figure S7c,d, display two linear regimes. The first occurs between 0 and 1 mM, exhibiting a sensitivity (*i.e.*, slope of the calibration curve) of 0.41 and 0.63 mA/(cm^2^·mM) for OMLP_f_/PHEDOT/PEDOT and OME_f_/PHEDOT/PEDOT, respectively. In the second regime, which extends from 1 to 6 mM, the sensitivity decreases slightly to 0.25 and 0.17 mA/(cm^2^·mM) for OMLP_f_/PHEDOT/PEDOT and OME_f_/PHEDOT/PEDOT, respectively. As the final aim of the coated meshes is the detection of NADH from bacterial activity, the critical range is the first one, as the NADH concentration values reported from bacterial cultures are lower than 1 mM.^31,47^ The presence of two linear regimes has been attributed to the complex geometry of the coated meshes, which affects the detection process with respect to simpler two-dimensional (2D) electrodes prepared by activating, functionalizing, and coating compact i-PP films.^31^ Thus, at high NADH concentrations, the access of the analyte molecules to the surface of film-shaped electrodes, which were found to exhibit a single linear regime,^31^ is much easier and robust than to the surface of complex mesh-shaped (*i.e.*, macroporous woven sheet) electrodes.

**Figure 8:**
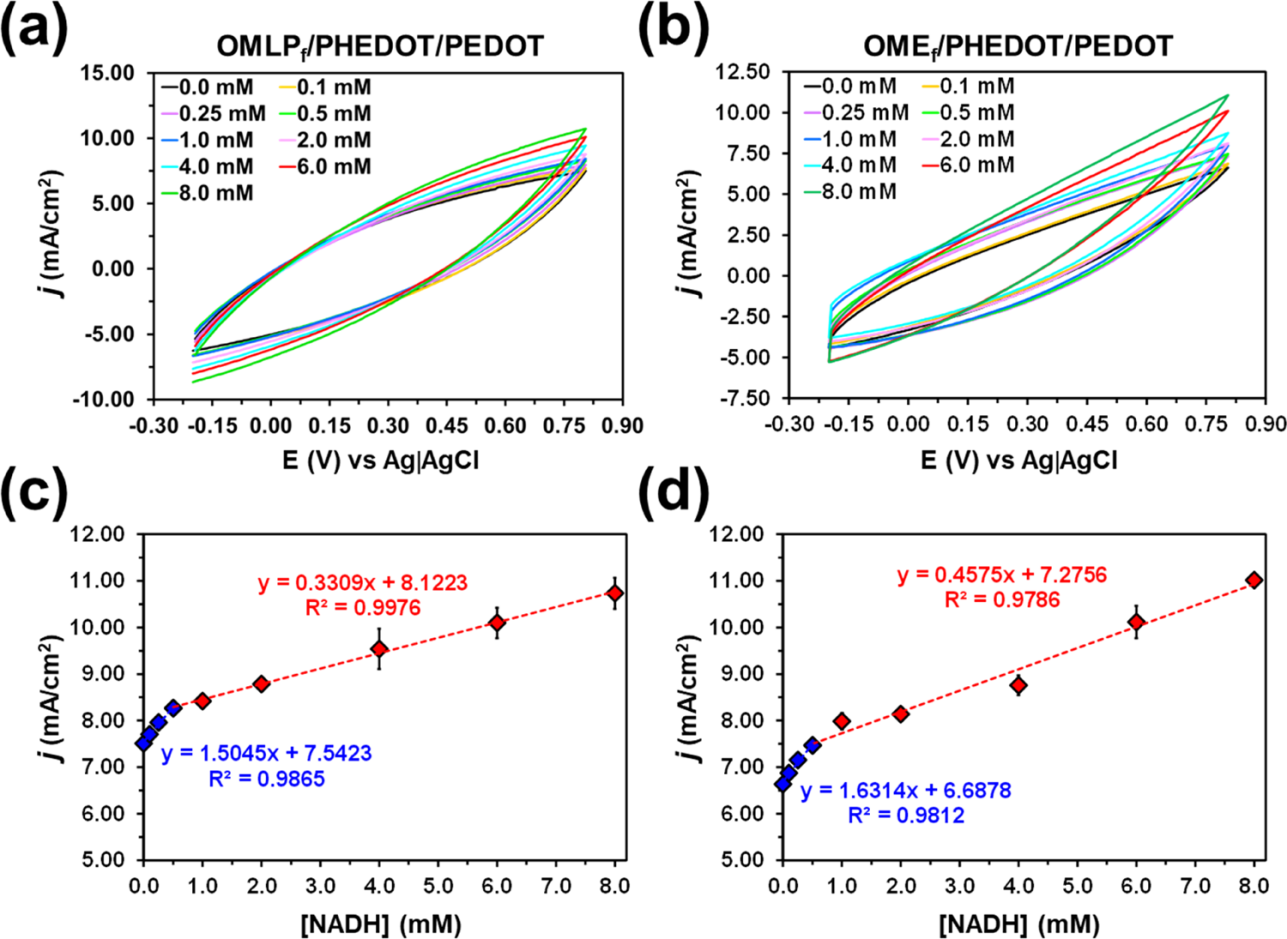
NADH detection: (a, b) cyclic voltammograms and (c, d) calibration profiles recorded for (a, c) OMLP_f_/PHEDOT/PEDOT and (b, d) OME_f_/PHEDOT/PEDOT. Voltammograms were recorded at a scan rate of 50 mV/s using 0.1 M PBS solutions at different concentrations of NADH.

To improve the electrochemical characterization of OMLP_f_/PHEDOT/PEDOT and OME_f_/PHEDOT/PEDOT electrodes for the detection of NADH, the scan rate used to record the voltammograms was systematically decreased from 100 to 25 mV/s. Results obtained using a scan rate of 75 mV/s were similar to those displayed in Figure S7 for 100 mV/s, whereas results obtained using a scan rate of 25 mV/s were very similar to those depicted in Figure 8a,b, which were achieved using a scan rate of 50 mV/s. As it is reported,^47,48^ the area of the voltammograms and *j*_max_ increased with decreasing scan rate, this behavior being similar for the two functionalized meshes. Furthermore, the voltammograms became more symmetric, especially for the OMLP_f_/PHEDOT/PEDOT. The results obtained indicated that at a lower scan rate (50 mV/s), an optimum equilibrium between the rate of diffusion and rate of reaction was reached, allowing a better detection. The calibration curves were obtained considering a higher number of NADH concentrations (*i.e.*, assays using 0.1, 0.25 and 8 mM NADH solutions were performed in addition to those employed for a scan rate of 100 mV/s) to be more precise (Figure 8c,d). Results evidenced that the effects attributed to the geometry of the mesh, as for example, the difference in the CY due to the different polymerization charge required, became less apparent with decreasing scan rate.

On the other hand, the detection limit (DL) has been defined as
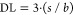
4where *s* is the standard deviation of the blank (*n* = 3) and *b* is the slope of the first linear regime. For OMLP_f_/PHEDOT/PEDOT and OME_f_/PHEDOT/PEDOT and a scan rate of 100 mV/s (50 mV/s), the DL values are 0.35 mM (0.09 mM) and 0.48 mM (0.18 mM), respectively. Considering the complex geometry and flexibility of i-PP meshes, these values are very satisfactory. Indeed, the DL values obtained using a scan rate of 50 mV/s are very similar to those reported for i-PP flat films (0.14 mM).^47^ Lower DL values have been recently reached using other sophisticated (semi)rigid electrodes, as, for example, nanoporous gold modified with diaphorase and osmium-based polymer (DL = 0.8 μM),^49^ aluminum hydroxide/iron hydroxide/MWCNTs nanocomposite (DL = 0.30 μM),^50^ and screen-printed electrode modified with reduced graphene oxide/polyneutral red/gold nanoparticles (DL = 0.38 μM).^51^ However, the DL values found for the sensor implemented in the surgical meshes described in this work are below the concentration of extracellular NADH determined for biofilm-forming and -nonforming bacterial cultures.^47^

### Electrochemical Detection of Bacteria Using Functionalized Surgical Meshes

In a recent study, the extracellular NADH, as determined by ultraviolet–visible (UV–vis) spectroscopy, was directly related to the quantity of bacteria in the medium, evidencing that such bioanalyte can be used to quantify the number of bacteria colonizing a mesh.^47^ In this work, the NADH present in bacteria (*S. aureus* and both B+ and B– *E. coli*) culture media was detected by CV using OMLP_f_/PHEDOT/PEDOT and OME_f_/PHEDOT/PEDOT as the working electrodes in an electrochemical sensor. Details of the experimental procedure are described in the Methods section. After promoting bacteria growth, the solutions were centrifuged, and the supernatant was frozen in liquid nitrogen and lyophilized until dry. The resulting powders were dissolved in 20 mL of milli-Q water, and this solution was consecutively diluted. Therefore, the sample solutions analyzed contained different concentrations of NADH coming from bacteria metabolism (*i.e.*, aerobic respiration reactions). As before, regardless of the functionalized mesh, the current density at the reversal potential (*j*_max_) increased with the concentration of NADH (Figure S8).

**Figure 9:**
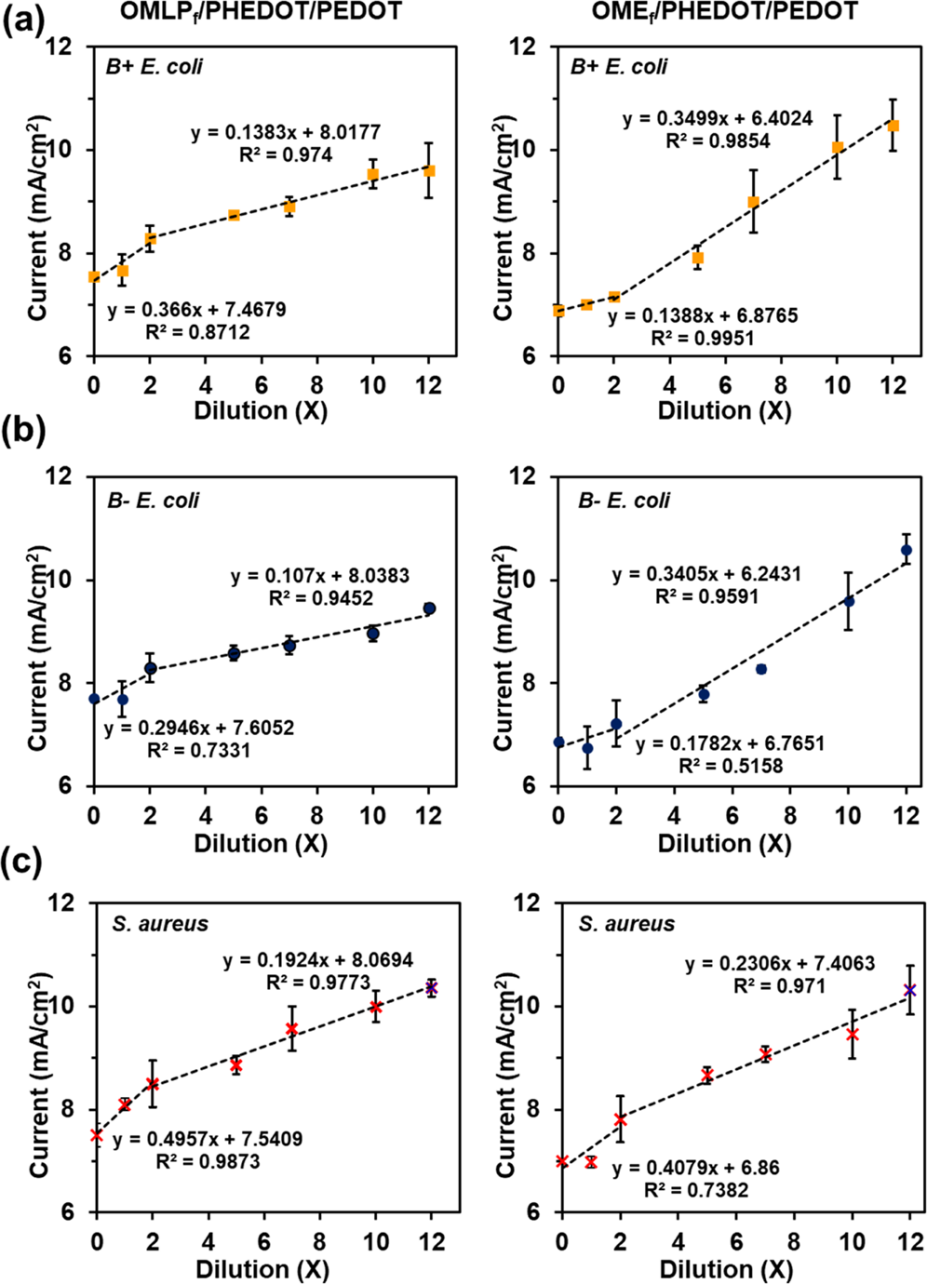
Electrochemical detection of bacterial NADH for OMLPf/PHEDOT/PEDOT (left column) and OMEf/PHEDOT/PEDOT (right column). Linear regression derived from the voltammograms displayed in Figure S8 (*n* = 3): (a) B+ *E. coli*, (b) B– *E.coli,* and (c) *S. aureus*.

**Figure 10:**
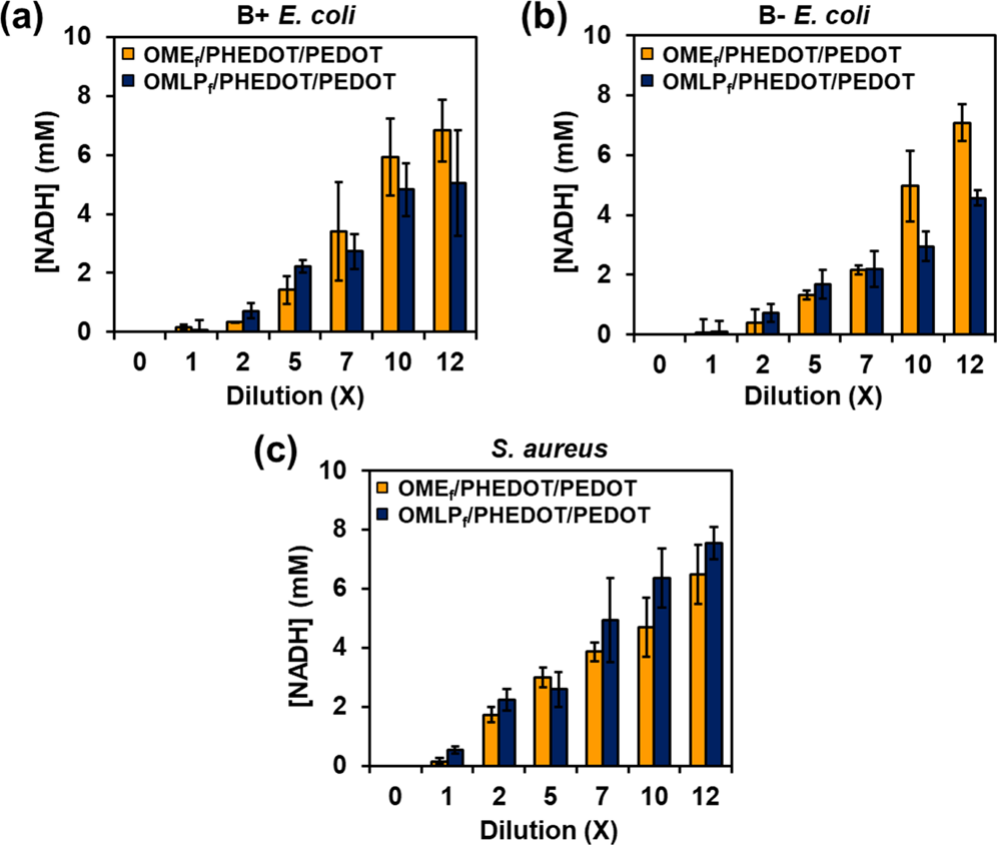
NADH concentration values (in mM) determined for each diluted bacteria culture medium solution using the calibration curves displayed in Figure S9 (*n* = 3): (a) B+ *E. Coli*, (b) B– *E. coli,* and (c) *S. aureus*.

Both OMLP_f_/PHEDOT/PEDOT and OME_f_/PHEDOT/PEDOT efficiently detected the presence of this bacterial metabolite, with data fitting a linear regression equation for each of the two regimes previously observed (Figure 9). By using the calibration curve obtained for the systems (Figure S9), the concentration of NADH was estimated for each bacteria culture dilution (Figure 10). Although in good agreement, values determined for each solution differ slightly depending on the mesh, which we ascribe to their distinct working area, as well as the complex geometry displayed by OME_f_/PHEDOT/PEDOT, which hinders to some extent the electrochemical detection (also seen in the calibration cyclic voltammograms, Figure S9).

Table 3 lists the NADH concentration values from B+ *E. coli*, B– *E. coli,* and *S. aureus* for OMLP_f_/PHEDOT/PEDOT and OME_f_/PHEDOT/PEDOT at 5× and 12× (Figure 10). As it can be seen, for a given dilution, the concentration of NADH is systematically higher for *S. aureus* than for B+ *E. coli*, which in turn is higher than for B– *E. coli*. Such ranking of NADH concentrations matches the values of the slope obtained for the calibration plots displayed in Figure 9 that, regardless of the mesh, exhibits the following order: *S. aureus* > B+ *E. coli* > B– *E. coli*. Overall, both functionalized meshes, and especially OMLP_f_/PHEDOT/PEDOT, can be applied as sensors to detect extracellular NADH from aerobic bacterial metabolism.

**Table 3:** NADH Concentration from B+ *E. coli*, B- *E. coli,* and *S. aureus* Cultures at 5× and 12× Dilutions^a^

mesh	dilution	B+ *E. coli*	B- *E. coli*	*S. aureus*
OMLP_f_/PHEDOT/PEDOT	5×	2.22 ± 0.206	1.67 ± 0.476	2.6 ± 0.6
	12×	5.05 ± 1.780	4.56 ± 0.248	7.6 ± 0.5
OME_f_/PHEDOT/PEDOT	5×	1.42 ± 0.479	1.32 ± 0.156	3.0 ± 0.3
	12×	6.83 ± 1.050	7.08 ± 0.610	6.5 ± 1.0

a Data derived from Figure 10.

## Conclusions

This work provides a procedure to modify surgical meshes so that, in addition to their functionality for supporting damaged tissue, they incorporate the possibility of detecting bacteria growth and, consequently, preventing post-operative infections on implanted medical devices. This procedure, which has been proved considering two different commercial surgical meshes, Optilene mesh LP and Optilene mesh elastic, consists of the following three steps: (1) activation with a plasma treatment to create oxygen-functional groups on the surface of the meshes; (2) functionalization of the activated meshes with PHEDOT NPs, which were prepared by oxidative chemical polymerization; and (3) coating of the functionalized meshes with a PEDOT layer, which was incorporated by electrochemical polymerization using the PHEDOT NPs as polymerization nuclei. Operational parameters for the latter electropolymerization process were optimized to get a balanced equilibrium considering the electrochemical response of the meshes, the homogeneity of the coating layer, and the handling capacity of the resulting sensor.

The two modified meshes were able to detect the oxidation of NADH to NAD+. It is worth noting that NADH is a metabolite from cell respiration reactions that leaves the extracellular space when it is produced by bacteria, but that remains in the cytosolic pool when it is produced by eukaryotic cells. Accordingly, the electrochemical detection of extracellular NADH is a powerful strategy to prevent bacterial infections in implanted meshes. *In vitro* studies have demonstrated the capacity of OMLP_f_/PHEDOT/PEDOT and OME_f_/PHEDOT/PEDOT to detect the dynamics of B+ *E. coli*, B- *E. coli* bacteria, and *S. aureus* activity by measuring the NADH from their respiration reactions. In summary, the modification of surgical meshes with CPs allowed us to add a new functionality, thus enabling the detection of bacteria growth through a specific metabolic analyte.

## Supporting Information Available

SEM and AFM images; Raman spectra; cyclic voltammograms; calibration plots and photographs (PDF)

## Notes

Authors declare the patent application for the electrochemical detection of bacteria using NADH as a biomarker (19-9288 EP).
