# Supplementary material for: Preparation and Characterization of Functionalized Surgical Meshes for Early Detection of Bacterial Infections
Source: ACS Biomater Sci Eng. 2023 Jan 24;9(2):1104–15. doi: 10.1021/acsbiomaterials.2c01319 (PMC10889595; doi:10.1021/acsbiomaterials.2c01319)
Supplement: Supplementary file 1 — ab2c01319_si_001.pdf [file ab2c01319_si_001.pdf]

SUPPORTING INFORMATION

Preparation and Characterization of Functionalized Surgical Meshes for Early Detection of Bacterial Infections

Adrián Fontana-Escartín,[1] Karima El Hauadi,[1] Sonia Lanzalaco,[1,] Maria M. Pérez-Madrigal,[1] Elaine Armelin,[1] Pau Turon,[2] and Carlos Alemán[1,3,*]*

[1] Departament d'Enginyeria Química and Barcelona Research Center for Multiscale Science and Engineering, EEBE, Universitat Politècnica de Catalunya, C/ Eduard Maristany, 10-14, 08019, Barcelona, Spain

[2] B. Braun Surgical, S.A.U. Carretera de Terrassa 121, 08191 Rubí (Barcelona), Spain

[3] Institute for Bioengineering of Catalonia (IBEC), The Barcelona Institute of Science and Technology, Baldiri Reixac 10-12, 08028 Barcelona Spain

Page S2	Table S1. Atomic composition of the studied meshes.
Page S3	Figure S1. Representative SEM micrographs of pristine and plasma activated OMLP meshes.<br>Figure S2. AFM image of pristine and plasma activated OMLP meshes.
Page S4	Figure S3. Raman spectra of pristine, activated, functionalized and coated meshes.
Page S5	Figure S4. Cyclic voltammograms and optical micrographs of OME_f/PHEDOT/PEDOT meshes prepared using a monomer concentration of 25 mM and different polymerization charges.
Page S6	Figure S5. Photographic images of pristine, plasma-treated, functionalized and coated meshes.<br>Figure S6. Cyclic voltammograms recorded for solutions with different $Fe(CN)_6^{3-}$ concentrations using functionalized meshes
Page S7	Figure S7. NADH detection using a scan rate of 100 mV/s: cyclic voltammograms and calibration profiles.
Page S8	Figure S8. Cyclic voltammograms recorded in bacteria culture medium at varying dilution from B+ *E. coli* and B- *E. coli* growth.
Page S9	Figure S9. Cyclic voltammograms recorded in DMEM supplemented medium with known NADH concentration values and calibration plots derived from the cyclic voltammograms.

Table S1. Atomic composition (in %) as determined by EDX spectroscopy of the activated, functionalized and coated meshes.

Mesh	C	S	O	N	Other
$OMLP_f$	76.6	-	3.8	19.5	0.1
$OMLP_f$/PHEDOT	77.0	0.3	6.0	16.6	0.1
$OMLP_f$/PHEDOT/PEDOT	60.2	14.5	18.2	5.2	1.9
OME_f	76.8	-	5.6	17.5	0.1
OME_f/PHEDOT	76.9	0.2	6.3	16.5	0.1
OME_f/PHEDOT/PEDOT	62.4	13.9	17.5	4.4	1.8

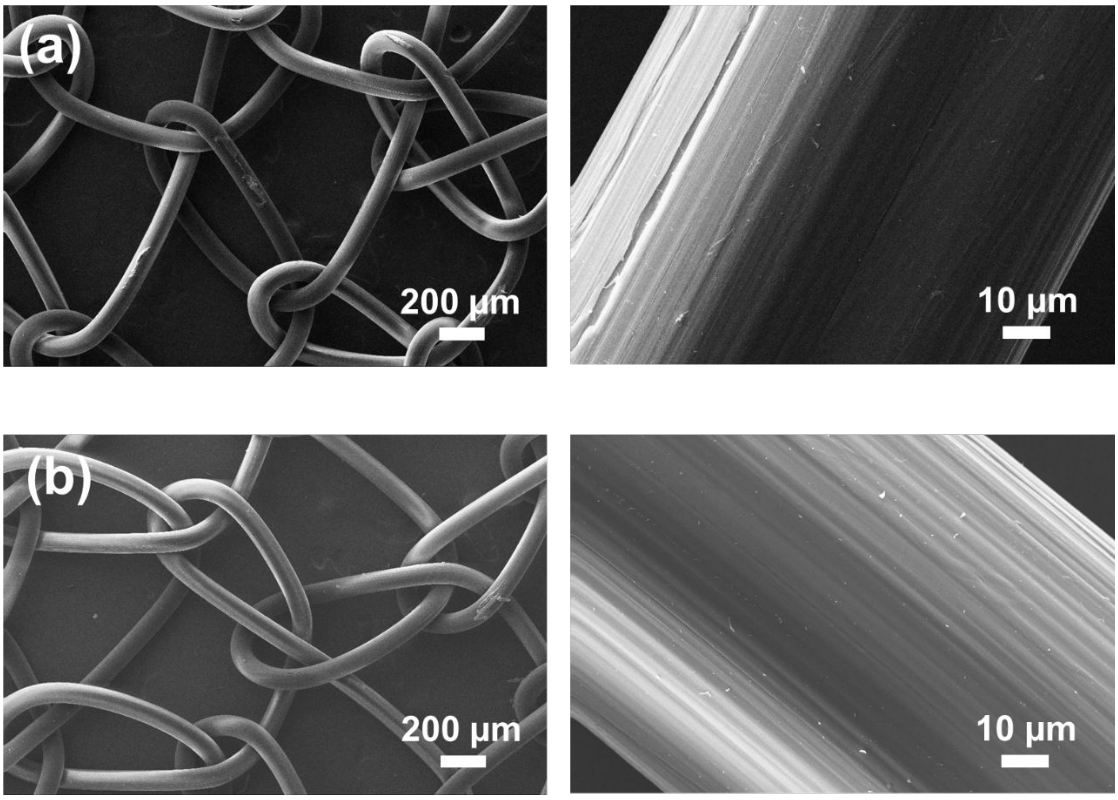

Figure S1. Representative SEM micrographs of (a) pristine and (b) plasma activated OMLP meshes. Low magnification micrographs of the mesh (left) and high magnification micrographs of the fiber (right) are displayed.

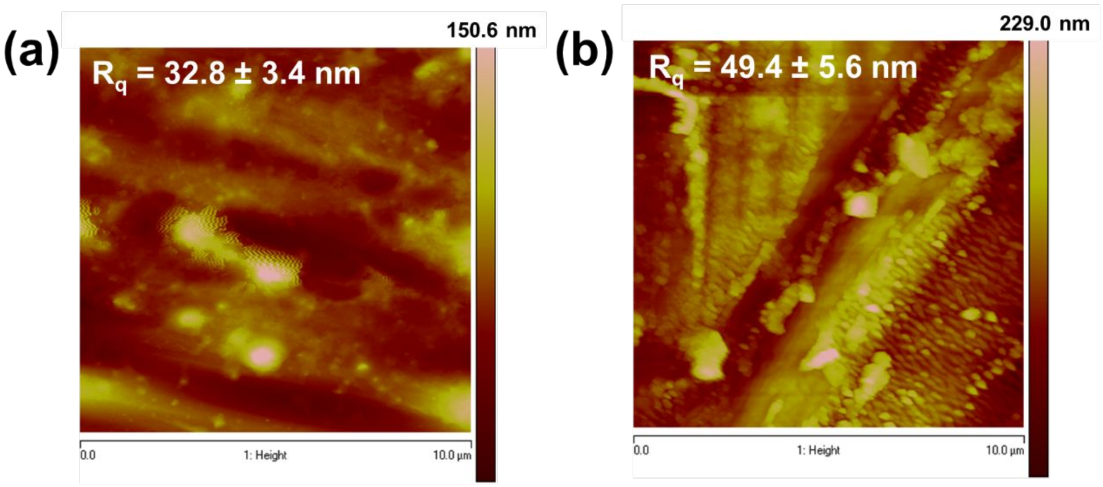

Figure S2. AFM image (10×10 μm^2) of (a) pristine and (b) plasma activated OMLP meshes. The root-mean-square roughness (R_q) is displayed for each system.

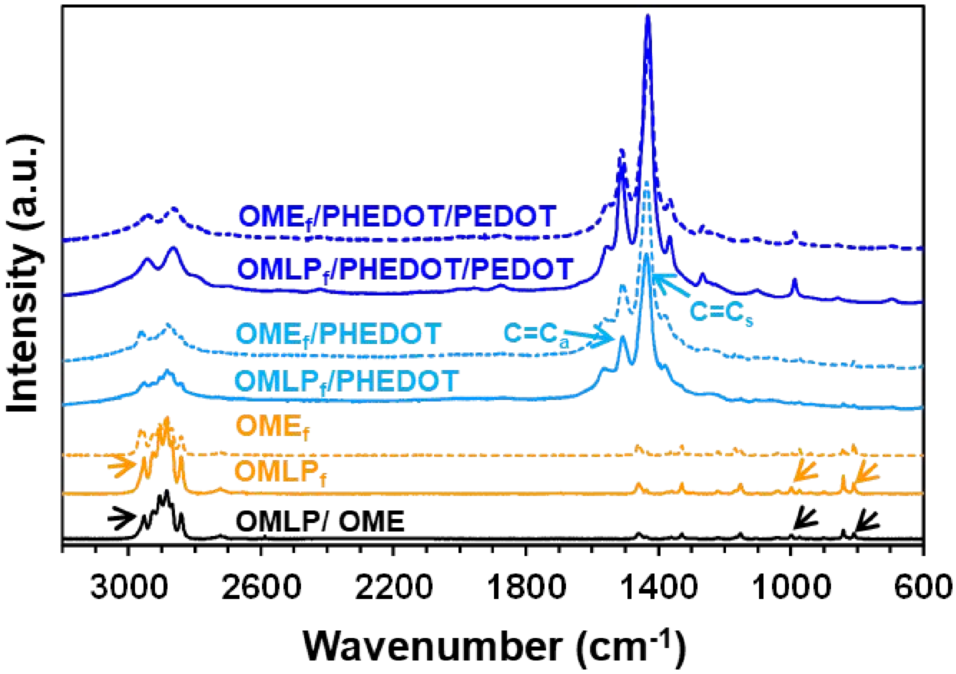

Figure S3. Raman spectra of pristine, activated, functionalized and coated meshes. Arrows indicate the bands that increased due to the plasma activation.

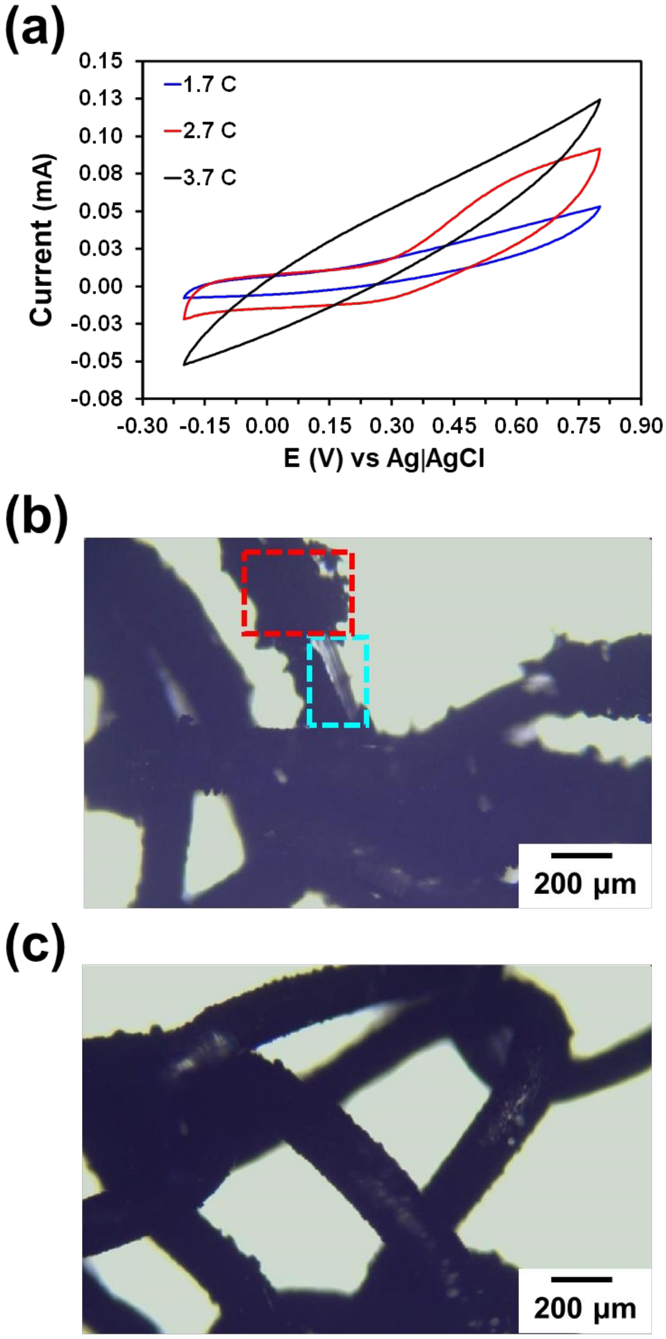

Figure S4. (a) Cyclic voltammograms of OME_f/PHEDOT/PEDOT meshes prepared using a monomer concentration of 25 mM and different polymerization charges (1.7, 2.7 and 3.7 C). The voltammograms displayed correspond to the 10th consecutive redox cycle and were recorded at a scan rate of 50 mV/s. Optical micrograph of OME_f/PHEDOT/PEDOT meshes prepared using: (b) a 25 mM EDOT concentration and a polymerization charge of 2.7 C, where the dashed red and blue boxes illustrate regions with an accumulation of PEDOT and a poor EDOT polymerization, respectively; and (c) a 25 mM EDOT concentration and a polymerization charge of 1.7 C, which shows a uniform PEDOT coating.

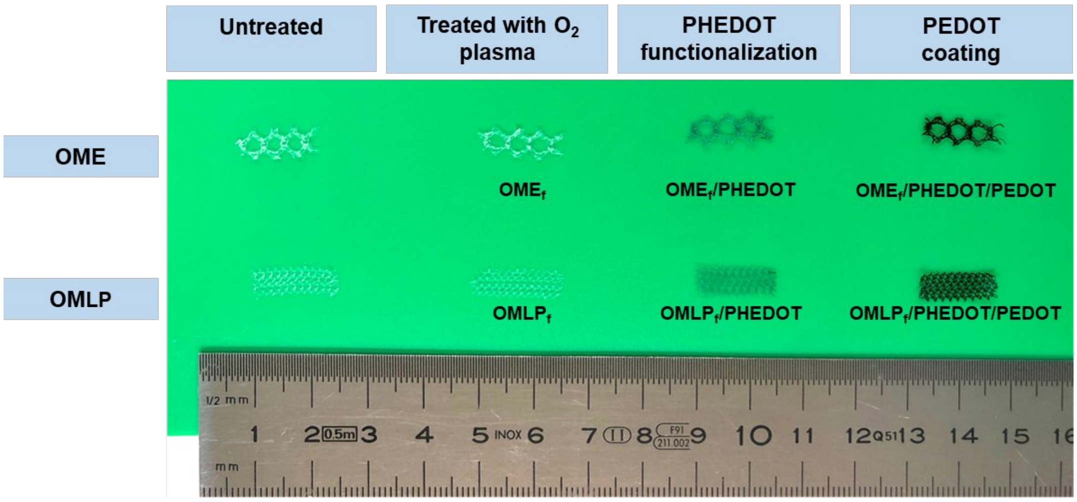

Figure S5. Photographic images of pristine, plasma-treated, functionalized and coated meshes.

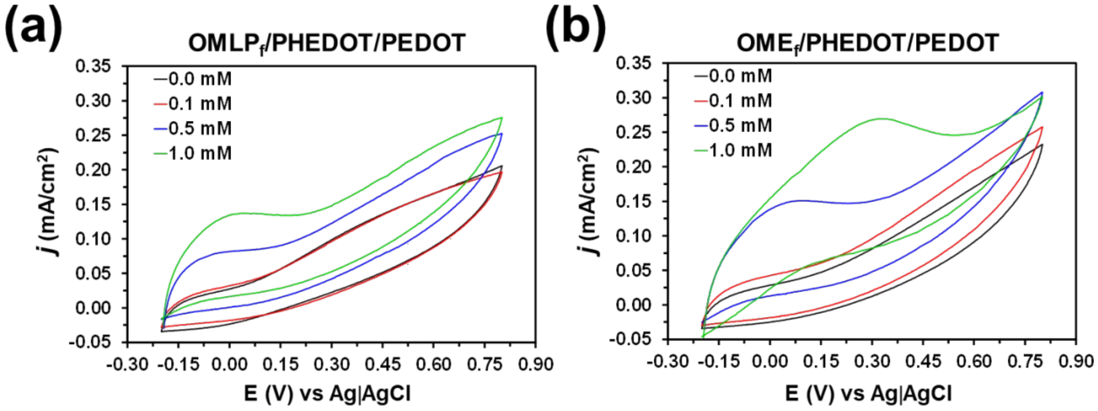

Figure S6. Cyclic voltammograms recorded for 0.1 M PBS solutions with different $Fe(CN)_6^{3-}$ concentrations (0.0, 0.1, 0.5 and 1.0 mM) using (a) $OMLP_f$/PHEDOT/PEDOT and (b) OME_f/PHEDOT/PEDOT meshes as working electrode. Scan rate: 50 mV/s.

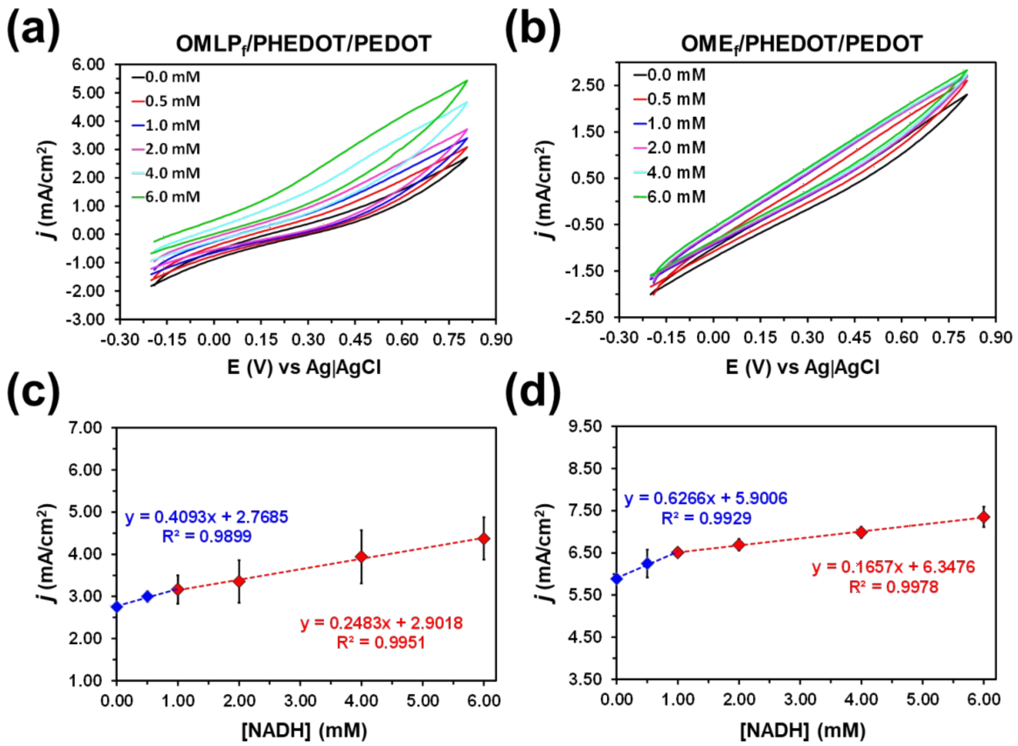

Figure S7. NADH detection: (a, b) cyclic voltammograms and (c, d) calibration profiles recorded for (a, c) $OMLP_f$/PHEDOT/PEDOT and (b, d) OME_f/PHEDOT/PEDOT. Voltammograms were recorded at a scan rate of 100 mV/s using 0.1 M PBS solutions at different concentrations of NADH.

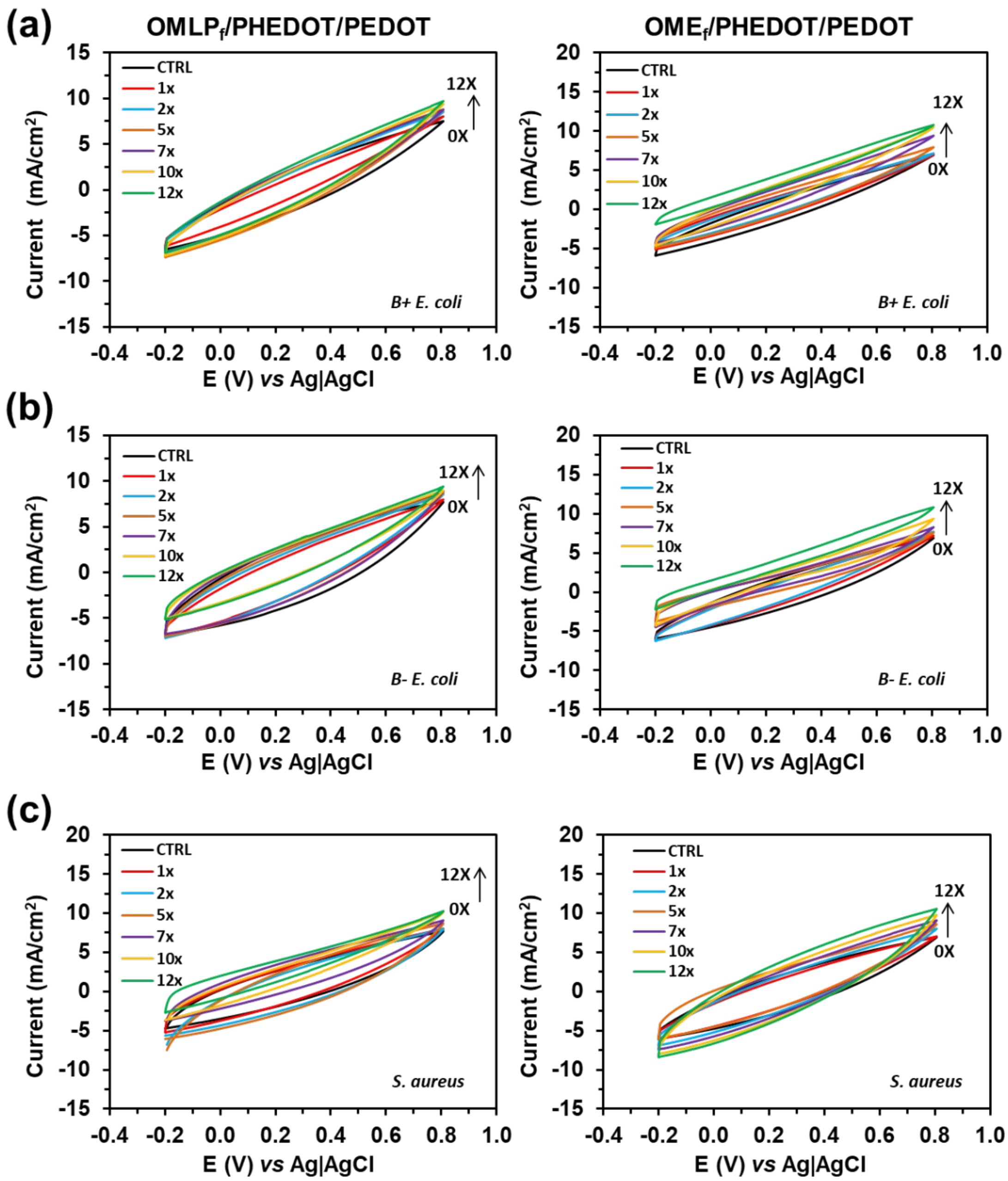

Figure S8. Cyclic voltammograms recorded in bacteria culture medium at varying dilution from (a) B+ *E. coli*, (b) B- *E. coli* and (c) *S. aureus* using OMLP$_f$/PHEDOT/PEDOT (left column) and OME$_f$/PHEDOT/PEDOT (right column) as working electrodes. Initial and final potential: −0.20 V; reversal potential: +0.80 V; Scan rate: 50 mV/s.

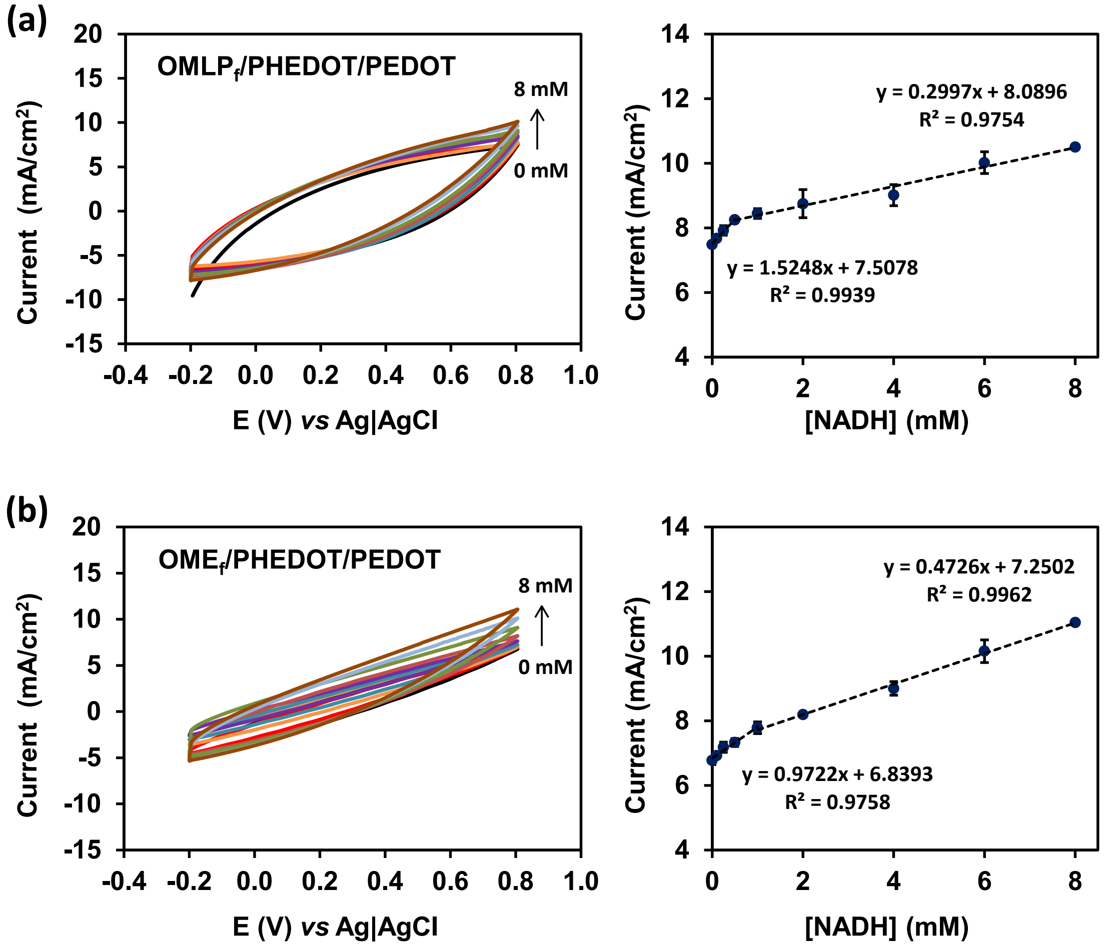

Figure S9. Left column: cyclic voltammograms recorded in DMEM supplemented medium with known NADH concentration values using (a) OMLPf/PHEDOT/PEDOT and (b) OMEf/PHEDOT/PEDOT as working electrodes. Initial and final potential: −0.20 V; reversal potential: +0.80 V; Scan rate: 50 mV/s. Right column: calibration plots derived from the cyclic voltammograms.
